# Odontogenic Keratocyst Presented as Multi-Locular Radiolucency in Mandibular Canine and Premolar Region: A Case Report

**DOI:** 10.7759/cureus.39291

**Published:** 2023-05-21

**Authors:** Abdulaziz Alwakeel, Mohamed Arakkal Vettath, Mohamed A Eltanany, Rayyan Waznah, Abdullah Aloufi

**Affiliations:** 1 Oral Medicine and Oral Pathology, Tabuk Dental Center, Tabuk, SAU; 2 Pediatric Dentistry, Tabuk Specialist Dental Center, Ministry of Health in Tabuk City, Tabuk, SAU; 3 Maxillofacial, Ain Shams University, Cairo, EGY; 4 Restorative Dentistry, Tabuk Dental Center, Tabuk, SAU; 5 Restorative Dentistry, Special Needs Dentistry, Tabuk Specialist Dental Center, Ministry of Health in Tabuk City, Tabuk, SAU

**Keywords:** dental procedures, oral pathology, unilocular lesion, odontogenic keratocyst, odontogenic cyst

## Abstract

An odontogenic keratocyst (OKC) was first described by Philipsen in 1956. They are benign cysts of odontogenic origin that behave aggressively and have a high recurrence rate.

The present case report describes an unusual presentation of OKC as a multi-locular lesion in the anterior mandible.

A 14-year-old male patient was referred to the oral maxillofacial surgery clinic in Tabuk Specialist Dental Centre by his orthodontist to evaluate a radiolucent lesion that had been identified in his lower anterior teeth during an OPG examination. The patient was medically fit and had multiple previous dental restorations. An intraoral examination revealed a small bony expansion in the cystic lesion on the buccal side. The panoramic radiograph showed well-defined multi-locular radiolucencies in the lower left canine area, despite there being no tooth resorption; however, there was a slight divergence noted between the teeth. An excisional biopsy was performed, and the subsequent histopathological examination revealed a cystic lesion diagnosed as an odontogenic keratocyst. The six-month follow-up OPG showed that the site had completely healed without any lesions recurring.

OKCs can present at any age, irrespective of gender. The differential diagnosis included a lateral periodontal cyst or a radicular cyst when the tooth was not vital. In this case, the six-month follow-up OPG following surgery revealed no recurrence, although a close follow-up is recommended because of the high recurrence rate.

## Introduction

An odontogenic keratocyst (OKC) was first described by Philipsen in 1956 [1]. They are benign cysts with an odontogenic origin that behave aggressively and have a high recurrence rate [2]. OKCs present twice as often in the mandibular arch as in the maxillary arch [3]. In the mandible, an OKC is usually present in the posterior area, which follows the angle and ramus of the mandible [4,5]. The latter OKC finding is root resorption, which is an uncommon radiographic feature with a reported incidence ranging from 1.3% to 11% [6].

Radiographically, OKCs usually appear as a well-defined unilocular or multilocular radiolucency bounded by corticated margins. Unilocular lesions are the predominant variant, whereas the multilocular variant is seen in approximately 30% of cases and mostly in the mandibular arch [6,7]. The most frequent genetic modification associated with OKC pathogenesis occurs in PTCH1 (Sonic Hedgehog (SHH) signaling pathway), and this has been detected in up to 93% of sporadic cases [8]. Interestingly, an activating mutation in the BRAF p.V600E gene, mainly related to ameloblastoma, although not the expression of its mutated protein product, has been reported in OKC [9].

In a systematic review, Johnson et al. showed that the patient treated by enucleation has the highest recurrence rate at approximately 30%, followed by marsupialization alone (approximately 18% recurrence rate) [10].

The present case report describes an unusual presentation of OKC as a multilocular lesion in the anterior mandible. 

## Case presentation

A 14-year-old male patient was referred to an oral maxillofacial surgery clinic in the Tabuk specialist dental center by his orthodontist to evaluate the radiolucent lesion that had been identified in his lower anterior teeth during the panoramic radiographic examination. The patient was medically fit, while their dental history revealed multiple dental restorations. No abnormality was identified in either the physical or additional oral examinations. An intraoral examination identified a little bony expansion in the area of a cystic lesion on the buccal side.

**Figure 1:**
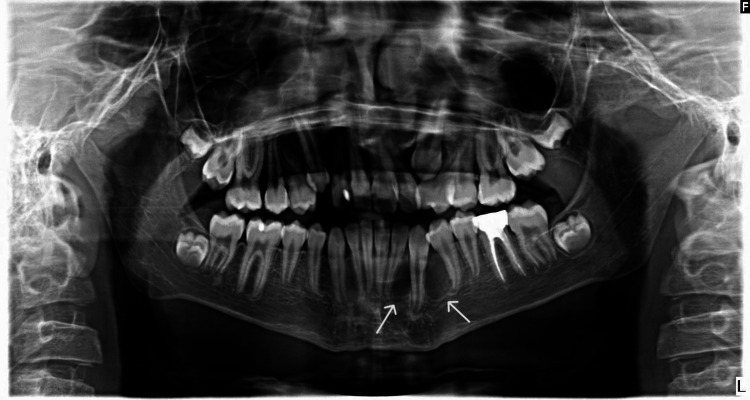
Panoramic radiograph shows multi-locular radiolucent surrounded lower left canine with a slight divergence between canine and premolar, lateral incisor.

The panoramic radiograph illustrated well-defined multi-locular radiolucencies in the lower left canine area, even though no tooth resorption was observed, although a slight divergence was noted between the teeth (Figure 1).

The differential diagnosis for this case included a lateral periodontal cyst, an odontogenic keratocyst, a botryoid odontogenic cyst, and an ameloblastoma.

**Figure 2:**
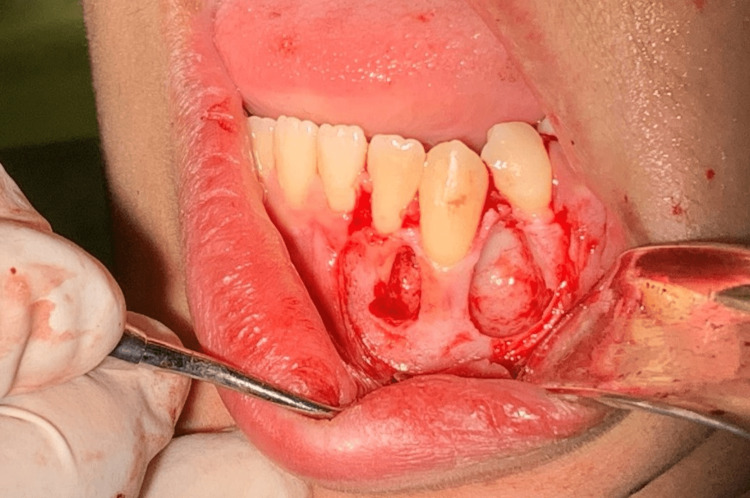
Intraoperative picture after removing the cystic lesion, showing two separated cystic spaces.

After discussing the possible diagnosis with the patient, the patient was referred for oral maxillofacial surgery for a biopsy, where an excisional biopsy was performed to confirm the diagnosis. The complications of the surgical procedure were discussed with the patient, and written informed consent was obtained from the patient. Next, an excisional biopsy was conducted under local anesthesia with 1.8 mL of anesthetic solution (lidocaine HCl 2% and epinephrine 1:100000) administered intraorally via an injection to the inferior alveolar and mental nerves of the left side of the mandible. The cystic lesion was enucleated by the surgeon and submitted for histopathological evaluation (Figure 2). The site of the surgery was closed using Vicryl triple zero sutures, and postoperative instructions were provided to the patient.

**Figure 3:**
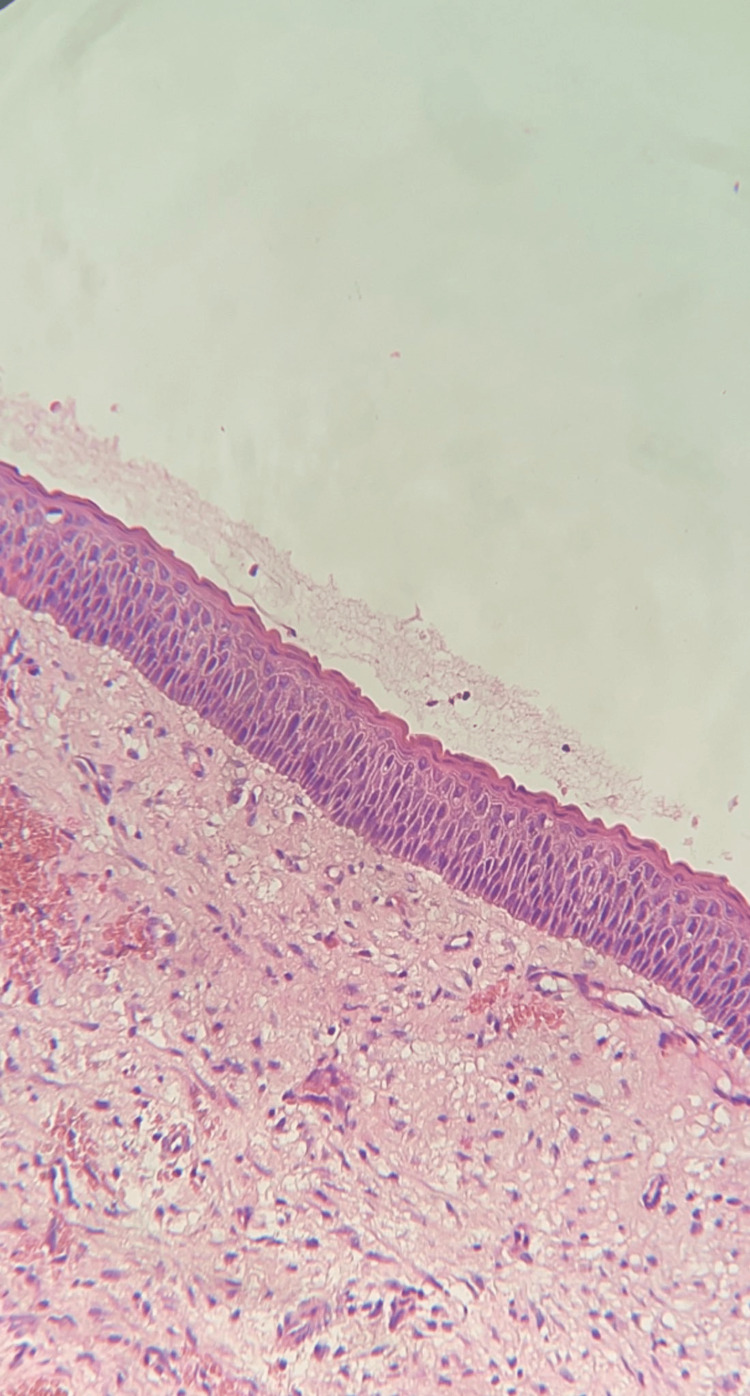
Histopathological examination revealed cystic lesion lining by para-keratinized stratified squamous epithelium with a corrugated pattern, 4–6 layers of cells, and peripheral palisading of the basement membrane basal cell layer.

The histopathological examination revealed a cystic lesion that was lined by para-keratinized stratified squamous epithelium, while the cystic lining was characterized as corrugated para-keratin with a thickness of 5-8 cells and basal layer peripheral palisading (Figure 3).

Treatment

**Figure 4:**
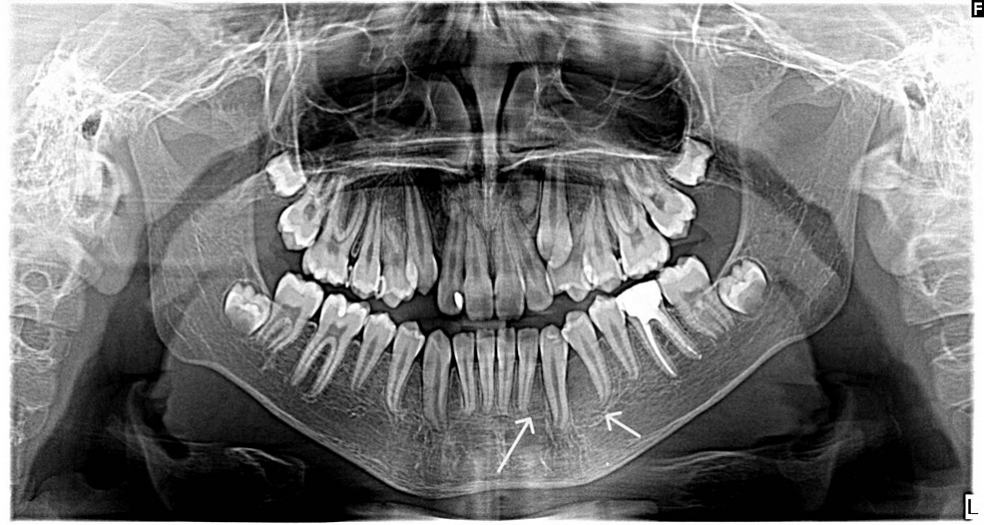
The panoramic radiograph showed complete healing at the surgical site without any recurrence after a six-month follow-up.

No further treatment was needed in this case. The outcome and follow-up clinical, radiographic, and histopathological examinations of the lesion were concluded to be OKC. The sixth-month follow-up panoramic radiograph showed complete healing at the surgical site without any recurrence (Figure 4).

## Discussion

The anterior maxilla, especially between the canine and lateral incisors, is the most common site for OKC, followed by the third molar region [11]. Large lesions are particularly observed at the angle and ramus of the mandible [12]. However, in this case, it was presented as a small multi-cystic lesion in the lower anterior area, lateral to the lower left canine. The molecular findings may change the methods used to manage patients diagnosed with OKCs by using open surgical and non-surgical options for their treatment [13].

Moreover, few or incomplete septa may be seen in unilocular OKCs using panoramic radiography, which are present in the mandibular region, although this finding is more common in larger cystic lesions. Approximately 30% of OKCs are usually associated with unerupted teeth, most commonly the third molars [14]. According to the literature, OKCs may be located in the periapical, pericoronal, or lateral root areas. In approximately 30% of cases, there was no relationship to dental structures [14]. However, in the present case, the lesion occurred as a small multi-locular lesion with no separation and was not associated with an unerupted tooth.

When a small unilocular OKC occurs in the anterior maxilla, a lot of differential diagnoses are considered, such as a radicular, lateral periodontal, or nasopalatine duct cyst [15]. In our case, it was a multi-locular lesion that was present in the anterior mandibular area. Odontogenic keratocyst could have been considered a differential diagnosis, along with lateral periodontal cyst and ameloblastoma.

The treatment options include several methods, including radical or conservative treatment. The radical methods include resection (marginal or en bloc), while the conservative treatments include simple cyst excision or marsupialization/decompression, which can be followed by a second surgery. In our case, the choice of treatment was the enucleation of the cystic lesion along with curettage [2]. Moreover, the patient had a follow-up after six months, where no signs of recurrence, either clinically or radiographically, were observed.

## Conclusions

OKCs can be presented in a wide age group with no sex priority. The most common location of OKCs is on the posterior side of the mandible. Conservative surgical excision can be a treatment option for small cysts. Close follow-up was recommended because the OKCs have a high recurrence rate. Our case showed no recurrence after six months of follow-up at the surgical site.
